# Adenosquamous Carcinoma of the Breast: Case Report and Literature Review

**DOI:** 10.7759/cureus.25940

**Published:** 2022-06-14

**Authors:** Shista Priyadarshini, Neha Singh, Haadi Ali, Vineela Kasireddy

**Affiliations:** 1 Internal Medicine, Guthrie Robert Packer Hospital, Sayre, USA; 2 Surgery, Post Graduate Institute of Medical Education & Research, Chandigarh, IND; 3 Internal Medicine, Geisinger Commonwealth School of Medicine, Scranton, USA; 4 Hematology and Oncology, Capital Region Medical Center, Jefferson City, USA

**Keywords:** screening mammogram, breast lump, indolent breast cancer, metaplastic breast cancer, adenosquamous breast carcinoma

## Abstract

Low-grade adenosquamous breast carcinoma (LGASC) is an atypical variant of metaplastic breast cancer. It differs from metaplastic carcinoma and has an indolent behavior. It usually presents as a palpable lump, unlike in our case, which had an incidental presentation. Because of its rarity, it often creates a clinical and diagnostic challenge. With the risk of local recurrence, the current management is aggressive with excision. Chemoradiation has been used in a few cases, but optimal management is unclear. Our manuscript aims to add to the existing knowledge on LGASC.

## Introduction

Low-grade adenosquamous carcinoma (LGASC) of the breast has been defined as a rare variant of metaplastic breast cancer [1]. Diagnosis is often challenging with its similarity to syringomatous adenocarcinoma. Given its rarity, the management of this entity is not standardized. We present a case of an incidental adenosquamous carcinoma (ASC) that was managed contrary to the current evidence and aimed to add to the existing literature.

## Case presentation

**Figure 1:**
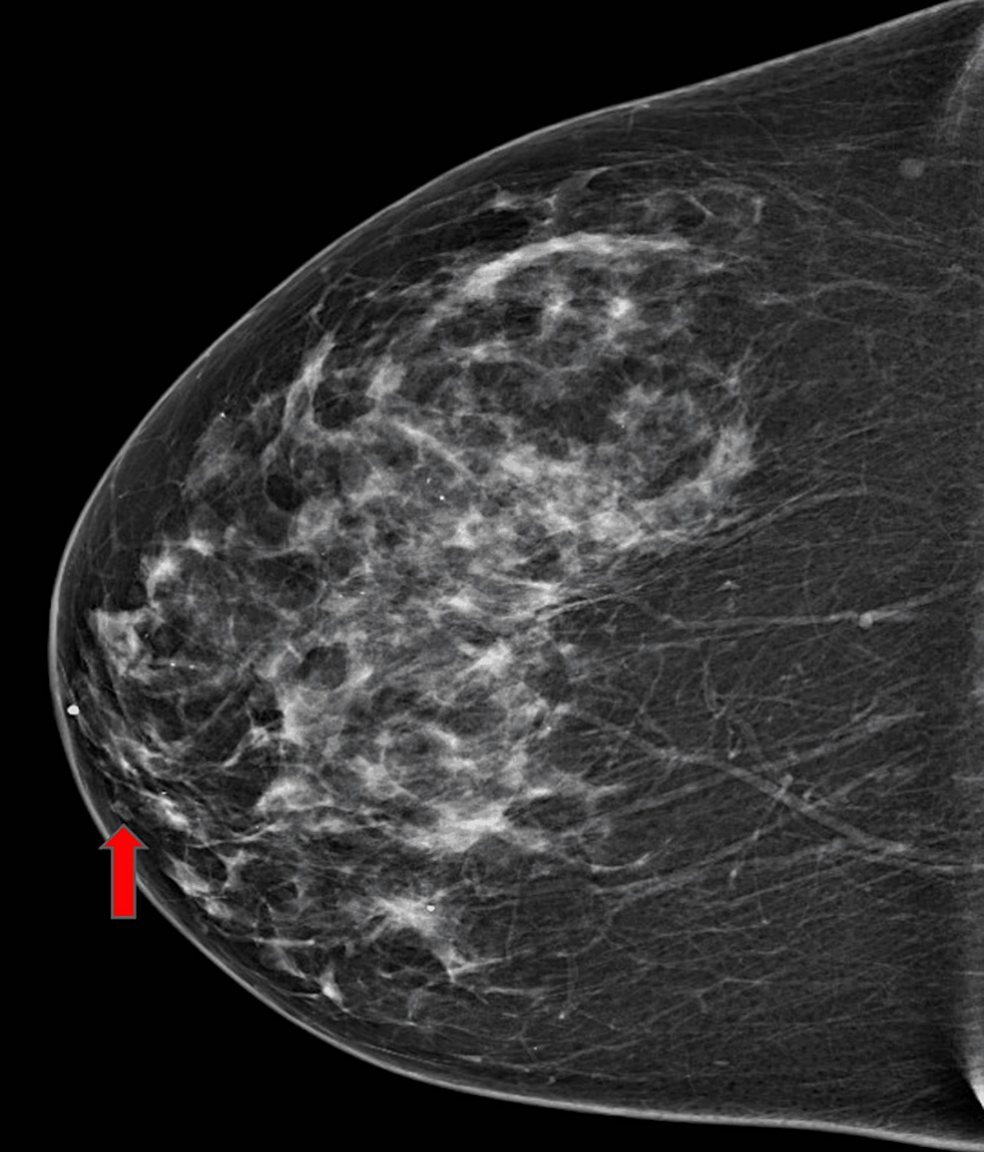
Screening mammogram showing hyperdense right breast and focal asymmetry (shown by red arrow)

A 72-year-old Caucasian woman presented for a wellness visit and denied any active complaints. Past medical history was significant for hypertension, hyperlipidemia, and type 2 diabetes mellitus. During the visit, she underwent a mammogram that showed focal asymmetry/distortion in the right breast (Figure 1). The screening mammogram was incomplete as bilateral breasts were dense and heterogeneous.

**Figure 2:**
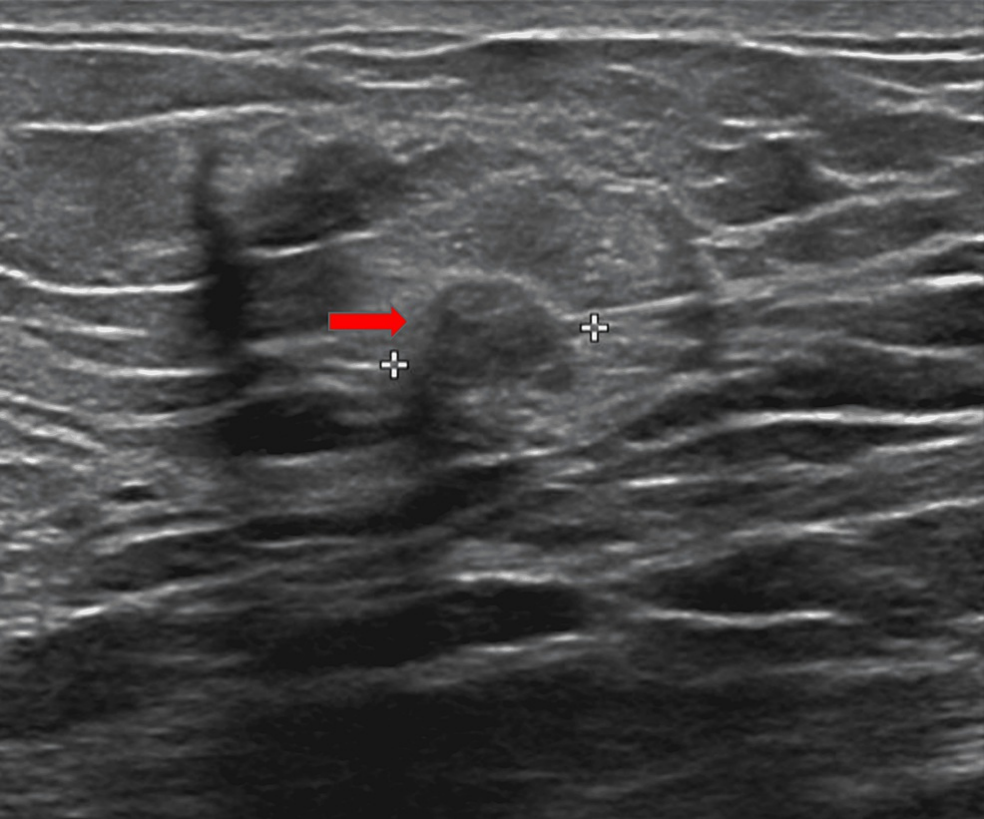
Ultrasound of right breast showing subareolar hypoechoic mass measuring 0.8 x 0.7 x 0.8 centimeter.

A three-dimensional breast tomosynthesis in craniocaudal and mediolateral oblique view was performed, which showed an 8mm subareolar hypoechoic mass in the right breast. This breast mass was graded as Breast imaging-reporting and data system (BI-RADS) category 4, suggesting the possibility of malignancy. A right breast ultrasound confirmed a right subareolar hypoechoic mass measuring 0.8 x 0.7 x 0.8 centimeter (Figure 2).

An ultrasound-guided core needle biopsy of the right breast showed fibro adipose breast tissue with tubular and squamous proliferation. The differentials included LGASC and syringomatous tumor, which would be difficult to differentiate with a superficial biopsy. The squamous components were negative for estrogen receptor (ER), progesterone receptor (PR), and human epidermal growth factor receptor 2 (HER-2) with a low proliferative index (Ki-67). The patient denied any fullness or pain in her breasts. A detailed physical examination showed no asymmetry, induration, discharge, mass, or palpable lymphadenopathy. Bilateral nipples were everted. A reflector localized right breast excisional biopsy showed LGASC, 10mm in greatest dimension with clear margins. As there is no clear role for adjuvant chemotherapy or radiation, she was observed with close surveillance after a multidisciplinary breast cancer conference. A 6-month follow-up showed architectural distortion at the site of previous partial mastectomy but no new masses or lymph nodes on advanced imaging.

## Discussion

ASC is rare and accounts for less than 0.2% of all breast cancer cases [1]. In 1912, Konjentzny first described adenosquamous findings in a breast tumor case. Rosen and Ernsberger first coined the term “LGASC” for breast in 1987 after studying and integrating eleven similar cases [2,3]. The World Health Organization (WHO) breast cancer classification in 2019 classifies ASC as a variant of metaplastic breast carcinoma. Metaplastic breast cancer has five other subtypes: spindle cell carcinoma, squamous cell carcinoma, metaplastic breast cancer with heterologous mesenchymal differentiation, fibromatosis-like metaplastic, and mixed metaplastic. Metaplastic breast cancer is an aggressive malignancy and constitutes 0.2-5% of all breast cancers [4]. It has a worse prognosis in comparison to non-metaplastic triple-negative breast cancer.

LGASC is an uncommon type of metaplastic breast cancer. Contrary to metaplastic breast cancer, LGASC has a more favorable prognosis. Microscopically, it is characterized by well-formed glands mixed with differentiated squamous cells in an abundant spindle cell stroma [5].

Most cases of LGASC present with palpable breast lumps of variable size, ranging from 2 to 5cm [6]. Their occurrence has been reported in women from 31 to 88 years of age. It usually has an indolent course with low metastasizing potential and an overall favorable prognosis [7]. These cancers are typically negative for ER, PR, and HER2 (triple-negative). Rare cases expressing estrogen receptor (ER) or progesterone receptor (PR) have been reported [8].

The most common benign lesion which mimics LGASC is sclerosing lesion. The LGASC diagnosis is supported by: glands invading adipose tissue, spindle cell stroma, triple-negative immunophenotyping, and myoepithelial marker variable staining. However, it is most challenging to distinguish between syringomatous adenoma and LGASC, which share many morphological findings. Both differ in location, with syringomatous adenoma presenting superficially, usually at the nipple dermis, while LGASC presents in deep breast parenchyma or peri-areolar area. Kawaguchi et al. described that syringomatous adenoma is unlikely to express core cytokeratin staining patterns and lamellar myoepithelial cuffing [9].

Classically metaplastic carcinoma is an aggressive malignancy, even though literature reports mammograms have benign findings [10,11]. Magnetic resonance imaging (MRI) typically presents an irregular and spiculated mass. A study in 2005 analyzed 12 cases of metaplastic breast cancer among a large cohort of 658 breast cancer cases, and MRI findings ranged from hypointensity or iso-intensity on T1 to hyperintensity on T2 [12]. So far, no characteristic mammographic or ultrasound findings have been reported for LGASC [13]. With limited findings on fine-needle aspiration and core needle biopsies, LGASC diagnosis is very challenging. The majority of cases are thus diagnosed after excisional biopsy [14].

There is currently no proposed optimal treatment for ASC, given its rarity. There is documentation of management ranging from local excision only to excision with 1 cm margins if lymph nodes are unaffected [3]. Since the current literature mentions local recurrence, it has led to the implementation of aggressive management with wide breast excision. However, regional lymph nodes (axillary lymph nodes) involvement and metastatic disease are very low. Given low incidences of nodal involvement, the patients could be managed with breast-conserving surgery [15]. Very few cases of LGASC have been documented with distant metastasis. It is often seen in large lesions, >3 centimeters in diameter, after repetitive local recurrences, or on transformation into a high-grade tumor [16]. Adjuvant chemoradiotherapy may be associated with improved survival only for tumors larger than 3 cm or confirmed lymphovascular invasion or nodal metastasis on histopathological examination. The current literature mentions just 1 case of local breast skin recurrence, which had a keloidal component and was managed with excision and close observation [17].

Survival in metaplastic breast carcinoma declines with advanced stage. Among patients managed with mastectomy and axillary lymph node dissection, the five-year survival rate ranges from 60-86% [18]. About half to two-thirds of all patients have local recurrence requiring local excision [19]. Contradictory to metaplastic carcinoma, LGASC is indolent and tends to local recurrence rather than distant metastasis. The 5-year survival rates in triple-negative ASC have been noted to be 81%, while in hormone-positive ASC, the rates decrease to 63% [1].

## Conclusions

LGASC is a distinct form of metaplastic breast cancer. Though it usually manifests as a palpable lump, it may be noted on an incidental mammogram. Diagnosis with imaging is challenging, and core needle biopsies may be used to rule out other malignant entities. Since there is no standardized management, the treatment approach can be individualized after assessing the size, extent, and lymphatic involvement. It usually has a favorable prognosis, and a breast conservation approach should be attempted when possible. Physicians should also closely monitor for local recurrence and very rarely nodal metastasis.
